# A prospective study of psychological distress among patients with advanced cancer and their caregivers

**DOI:** 10.1002/cam4.5713

**Published:** 2023-03-19

**Authors:** Irene Teo, Sean Ng, Filipinas Gines Bundoc, Chetna Malhotra, Semra Ozdemir, Jennifer L. Steel, Eric A. Finkelstein, COMPASS Group

**Affiliations:** ^1^ Lien Centre for Palliative Care, Duke‐NUS Medical School Singapore Singapore; ^2^ Signature Programme in Health Services and Systems Research, Duke‐NUS Medical School Singapore Singapore; ^3^ National Cancer Centre Singapore Singapore Singapore; ^4^ Saw Swee Hock School of Public Health, National University of Singapore Singapore Singapore; ^5^ Department of Surgery University of Pittsburgh School of Medicine Pittsburgh Pennsylvania USA; ^6^ Duke Global Health Institute, Duke University Durham North Carolina USA

**Keywords:** clinical cancer research, epidemiology, psychosocial studies, quality of life

## Abstract

**Background:**

Cancer can impact the psychological well‐being of both patients and their informal caregivers. We investigated the joint trajectories of psychological distress among Singaporean advanced cancer patients–caregiver dyads. We also examined predictors of trajectory group membership.

**Methods:**

This study utilised data from 299 patients with advanced solid cancer and their caregivers over 33 months (12 times points). Group‐based trajectory modelling was used to examine the joint trajectories of patient anxiety, patient depression, caregiver anxiety and caregiver depression scores using the Hospital Anxiety and Depression Scale.

**Results:**

Four joint trajectory groups were found: (1) Patient–caregiver low distress (27%), (2) patient–caregiver increasing distress (28.5%), (3) patient low‐ caregiver borderline distress (25%), (4) patient‐caregiver high distress (19.5%). Dyads where the patient is below 50 years of age were more likely to be in Group 4. Dyads where caregiver–patient emotional closeness was low were more likely to be in Groups 2 or 4 where dyads reported increasing/high distress. Dyads that reported financial inadequacy were more likely to be in Groups 2, 3 and 4, while dyads with caregivers who were employed were more likely to be in group 3.

**Conclusions:**

A substantial proportion of patients and caregivers reported anxiety and/or depression that lasted or increased throughout the study duration. We found significant heterogeneity in how dyads experienced psychological distress, suggesting that efforts should consider dyadic differences when providing psychological support. Particular focus should be placed on identifying dyads that are at risk and who require additional support.

## 1 INTRODUCTION

Cancer is a leading cause of death in many developed countries.^1^, ^2^ While patients report serious physical and psychological sequelae,^3^, ^4^, ^5^ the cancer burden is also borne by their informal, family caregivers (hereafter referred to as ‘caregivers’). Caregivers of cancer patients report financial struggles, poor sleep quality and psychological sequalae, with nearly half experiencing psychological distress (defined as having anxiety and depression symptoms).^6^, ^7^, ^8^ These concerns are likely to be amplified with lengthening patient survival^9^ and increasing outpatient care.^10^

It is very likely that patients and caregivers react to cancer‐related stressors interdependently.^11^ Evidence for these interdependencies were found in a review underlining complementary relationships between the psychological outcomes of dyadic members (e.g. higher caregiver anxiety was associated with greater patient depression).^12^ This suggests that the well‐being of dyads is intricately linked, underlining the importance of identifying factors contributing to dyadic psychological stressors.

Despite evidence of psychological interrelationships between dyads, longitudinal studies of distress have focused independently on patients or caregivers.^13^, ^14^, ^15^, ^16^ For instance, Park et al. examined the distress trajectories of breast cancer patients over 12‐month post adjuvant therapy and found two distinct patterns of distress—a ‘low‐decreasing distress’ group that showed minor improvements over time and a ‘consistently high‐distress’ group.^13^, ^17^ Similar patterns were found in studies examining caregivers—Sand et al. reported distinct subgroups of caregivers that showed distress trajectories that remained consistently low/moderate, decreased over time and remained consistently high/increased among parent caregivers one‐year post child–patient's haematopoietic stem cell transplant.^15^ Despite these studies, little is known regarding distress trajectories of advanced cancer patient‐caregiver dyads, especially in Asian cultural settings.^18^, ^19^ Examining patterns of dyadic distress over time can provide a deeper understanding of distinct distress trajectories. This can allow interventions to better recognise and support those at risk.

Literature examining the predictors of psychological distress among cancer patients and their caregivers suggest that information like patient/caregiver age,^20^, ^21^ gender,^22^, ^23^ marital status,^24^, ^25^ financial adequacy,^26^, ^27^ employment status,^28^, ^29^ relationship with one another (i.e. spouse and child)^30^ and emotional closeness^31^ are important characteristics to consider, especially in different cultural settings as they can differ across societal norms. For example, those from an Asian cultural background are likely to adhere to traditional gender roles and be more family‐oriented in their disease coping.^32^

This study aimed to describe the joint trajectories of psychological distress in a sample of patients with advanced solid cancer and their caregivers over a period of 33 months in Singapore. This study also examined the predictors of joint trajectory group membership—we hypothesised that patient and caregiver age and gender, as well as caregiver marital status, working status, financial adequacy, relationship (e.g. adult, child and spouse) and emotional closeness with patient would predict group memberships.

## 2 METHODS

### 2.1 Study design and participants

The present study utilised patient‐caregiver dyad data from the ‘Cost of Medical Care of Patients with Advanced Serious Illness in Singapore’ (COMPASS) study, an ongoing Singapore cohort study following advanced cancer patients and their informal caregivers.^33^ The COMPASS study enrolled 600 patients with solid, metastatic stage IV cancer who were/are followed up every 3 months until death. Participants were recruited from outpatient clinics at medical oncology departments of two major Singaporean public hospitals from July 2016 to May 2021. This study included data from baseline to month 33 (12 time points) of dyads who answered each survey within 14 days of each other.

The inclusion criteria for patients included: stage IV solid malignancy; aged ≥21 years; Singapore citizens or permanent residents; functional performance status on the Eastern Cooperative Oncology Group of ≤2 at baseline (to allow sufficient period of follow‐up); cognitively able to participate, which was determined through medical records and Abbreviated Mental Test administered to participants ≥60 years old. Caregiver participants were included if they were one of the main persons: (i) providing care to the patient; (ii) ensuring provision of care to the patient (e.g. hiring of paid caregivers; transport to medical appointments); (iii) involved in treatment decisions on the behalf of the patient. Foreign domestic workers (who are frequently hired to care for patients in Asian countries) were excluded. Both patients and their caregivers who agreed to participate provided written informed consent. The COMPASS study was approved by SingHealth Centralised Institutional Review Board (2015/2781). Study details are published elsewhere.^33^

### 2.2 Measures

#### 2.2.1 Patient and caregiver characteristics

Patients' and caregivers' age, gender and highest educational attainment were queried through a self‐report questionnaire. Caregivers were also asked to report their marital and employment status, relationship with patient, perceived financial adequacy and emotional closeness to patient. Refer to Table 1 for more information.

**TABLE 1:** Patient and caregiver characteristics (*N* = 299 dyads)

	Patient *n* (%)	Caregiver *n* (%)
Socio‐demographic characteristics		
Age, mean (SD)	61.6 (10.7)	49.3 (14.4)
Gender		
Female	157 (53%)	192 (64%)
Male	142 (47%)	107 (36%)
Ethnicity		
Chinese	227 (76%)	223 (75%)
Malay	52 (17%)	51 (17%)
Others	20 (7%)	25 (8%)
Highest education (years of education)		
Primary or lower (≤ 6)	124 (42%)	46 (15%)
Secondary (≤ 10)	86 (29%)	85 (29%)
Above secondary (> 10)	89 (30%)	167 (56%)
Religion		
Christianity	67 (23%)	71 (24%)
Islam	57 (19%)	59 (20%)
Free thinking/no religion	52 (17%)	53 (18%)
Others (Buddhist/ Taoist/ Hindu/ Sikh)	123 (41%)	116 (38%)
Married	245 (82%)	236 (79%)
Working full−/part‐time	97 (32%)	179 (60%)
Relationship to the patient		
Spouse		153 (51%)
Child		107 (36%)
Others		39 (13%)
Other caregiver‐reported characteristics		
Perceived adequacy of financial resources^a^		
Adequate/more than adequate		196 (66%)
Occasionally adequate		59 (20%)
Usually inadequate		38 (13%)
Living with the patient		232 (78%)
Received help with caregiving tasks		204 (68%)
Emotional closeness to patient		
Not at all close		2 (1%)
A little bit close		15 (5%)
Quite close		98 (33%)
Very close		183 (61%)

Abbreviation: ECOG, Eastern Cooperative Oncology Group performance score.

^a^ Does not add up to 100 due to missing data.

#### 2.2.2 Psychological distress

Patients' and caregivers' psychological distress (anxiety and depression) was measured using the Hospital Anxiety and Depression Scale (HADS), an instrument that has been reported to be reliable and valid for use in Singapore.^34^, ^35^ The HADS contains 14 self‐reported items scored on a 4‐point Likert scale (0–3). The Anxiety (HADS‐A) and Depression (HADS‐D) subscales are represented by the sum of 7 items each.^34^ A score of ≥8 on either subscale is suggestive of the presence of anxiety or depressive symptoms, respectively.

#### 2.2.3 Emotional closeness

Emotional closeness was assessed with the question: ‘How emotionally close are you to the patient?’. Responses were examined on a 4‐point Likert scale (1 – 4), with higher scores representing higher levels of emotional closeness.

### 2.3 Statistical analysis

Our study sample included patients and their caregivers who answered the survey from baseline to the 33rd month of follow‐up (or until patient death) and who answered each survey less than 14 days apart. We used multi‐outcome group‐based trajectory models (GBTM) to assess the heterogeneity in patterns of change in patient–caregiver dyad psychological distress (HADS‐A, HADS‐D). The joint trajectories consisted of the following continuous score outcomes: patient anxiety, patient depression, caregiver anxiety, caregiver depression. Group‐based trajectory modelling (GBTM) is a statistical method that identifies latent groups of patient–caregiver dyads who have similar joint trajectories over time for outcomes of interest. Group‐based *multi*‐trajectory modelling, an extension of GBTM, allowed us to jointly model the joint trajectories of anxiety and depression scores of *both* patients and caregivers. The model uses full‐information maximum likelihood to handle missing data, which includes after patient death.

We modelled patient‐caregiver dyad psychological distress assuming a censored normal distribution. We tested a varied number of models consisting of 1–5 trajectory groups and different polynomial functions for each trajectory from quintic to intercept. We systematically tested a series of model specifications changing the number of trajectory groups and polynomial specifications each time. To select the best fitting model of trajectory groups of psychological distress over time, we used Bayesian information criterion (BIC), value of trajectory membership probability (at least 5%) and of average posterior probability (threshold: 0.7) as our criteria.^36^

After choosing the best‐fit model, we tested the potential predictors of group membership using multinomial logistic regressions. Included predictors were patient socio‐demographic characteristics (age and gender) and caregiver characteristics (age, gender, marital status, employment status, financial adequacy and relationship with the patient). All analyses were conducted using Stata 15.1.

## 3 RESULTS

Data from 299 patient–caregiver dyads were analysed (see supplementary Figure S1). The demographic characteristics of the patients and caregivers are reported in Table 1. Patients had a mean age of 61.6 years (SD = 10.7), majority were female (53%), Chinese (76%) and plurality (42%) had up to a primary school‐level education. Caregivers had a mean age of 49.3 years (SD = 14.4), with majority being female (64%) and spouses of patients (51%). 94% of patients were at ECOG 0 at study baseline. Majority of caregivers had attained post‐secondary school education (56%) and reported adequate or more than adequate financial resources (66%). Refer to Table 1 for further patient and caregiver characteristics. At the end of the 33 months, 58% of patients had passed on (see supplementary Table S1).

### 3.1 Patient and caregiver distress at baseline

**FIGURE 1:**
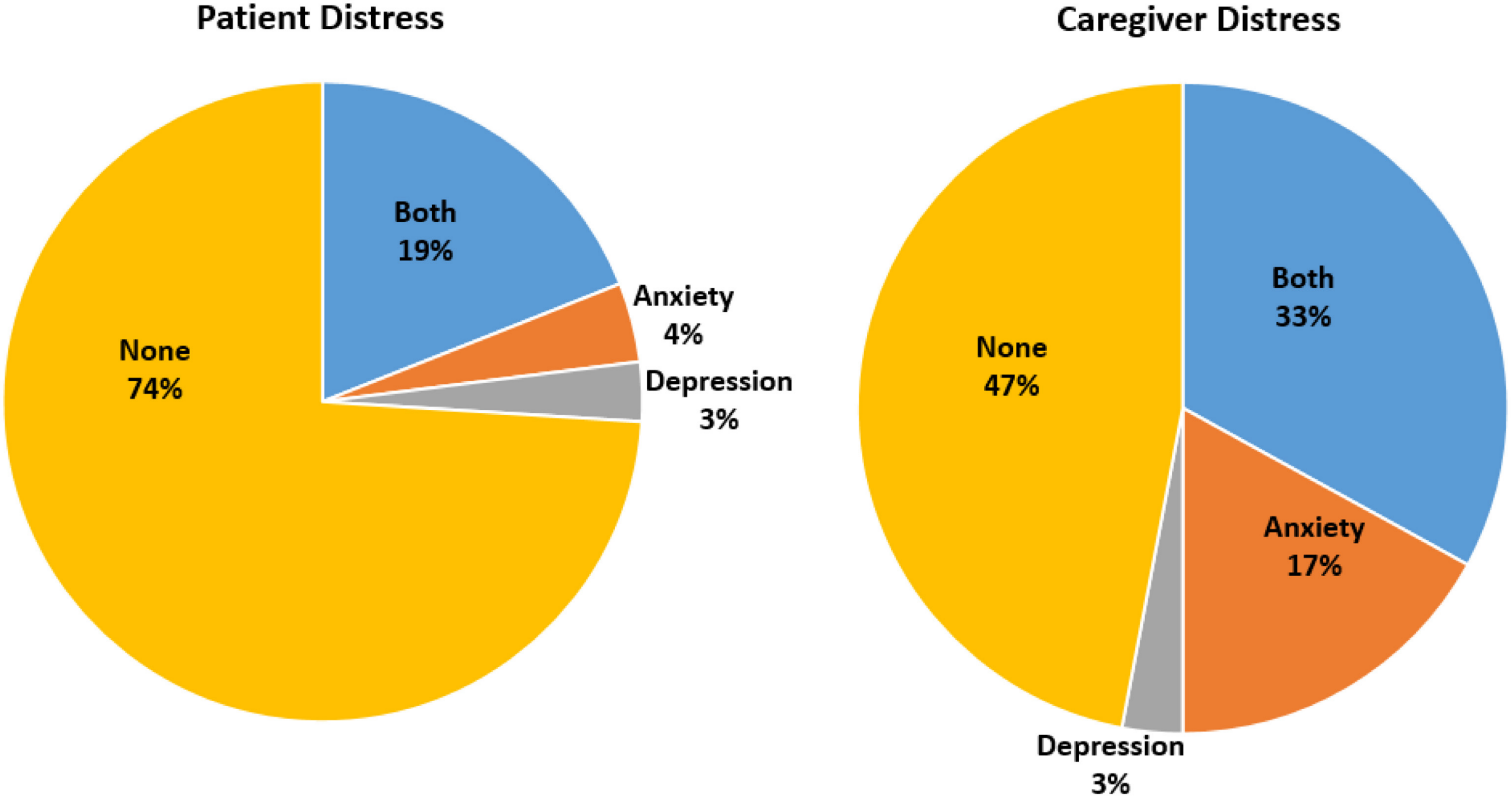
Patient and caregiver distress at baseline. Anxiety and depression were assessed using the Hospital Anxiety Depression Scale (HADS) threshold score of ≥ 8 respectively.

The majority (74%) of patients reported baseline scores below the threshold for distress (score of 8 for HADS‐A and HADS‐D, respectively) (see Figure 1). Of those who reported scores indicating distress, the majority (19% of all patients) met the threshold for both anxiety and depression. Few patients met the thresholds for only anxiety (4%) or only depression (3%). A different pattern was seen for caregivers, where more than half (53%) of caregivers reported scores indicative of distress: 33% met the threshold for both anxiety and depression, while 17% met thresholds for only anxiety and 3% only depression.

### 3.2 Patient–caregiver dyad trajectory groups

**FIGURE 2:**
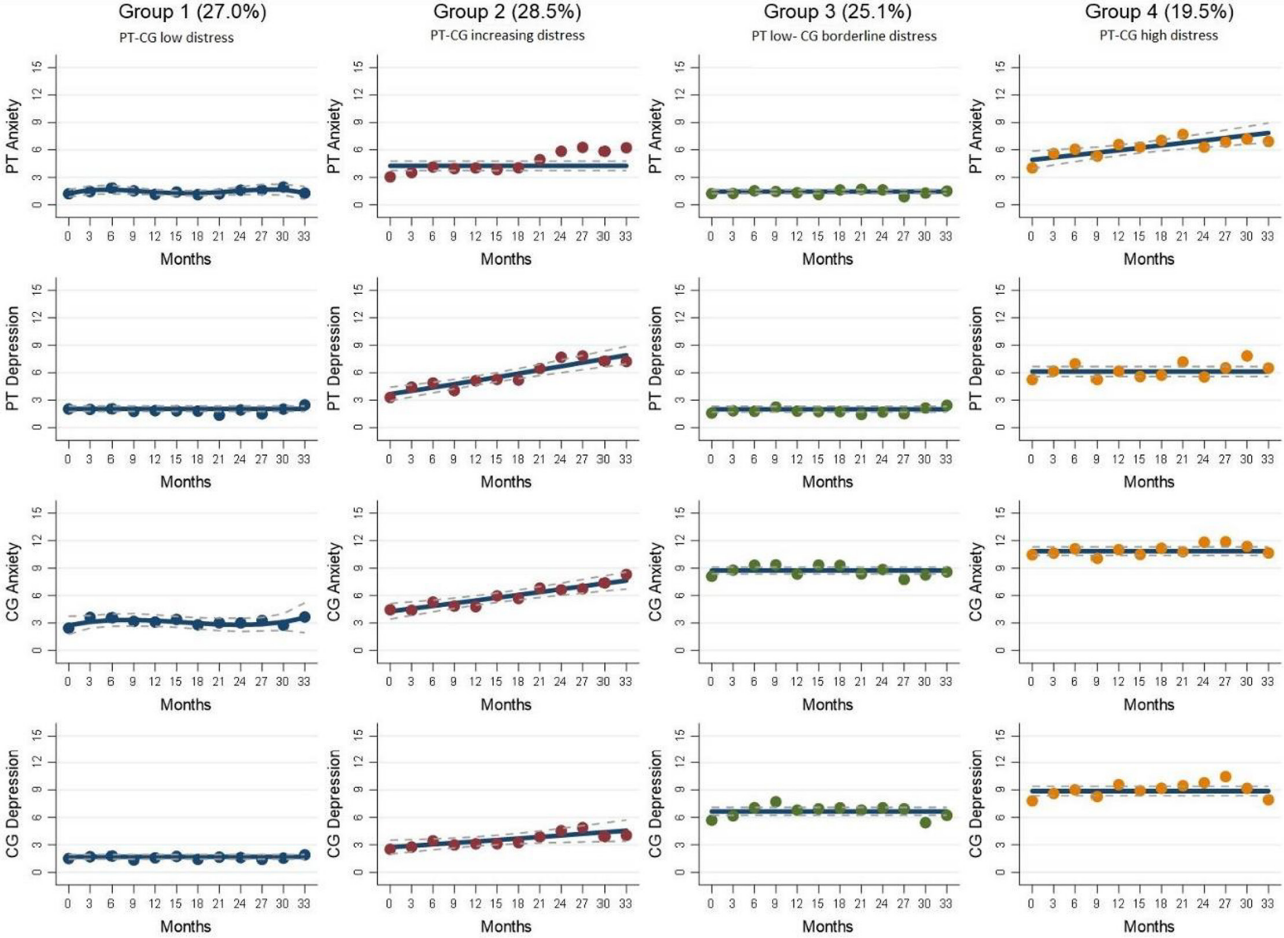
Psychological distress joint trajectories of 299 patient–caregiver dyads across 33 months. PT, patient; CG, caregiver.

We fitted 47 models with varying number of trajectory groups and functional forms to select the best fitting model (see supplementary Tables S1 and S2). We selected a four‐group trajectory model with BIC: −16435.57, average posterior probabilities of trajectory membership: 0.86 to 0.95 (Figure 2). We chose this model considering statistical fit of the model and parsimony. Because the % change in BIC from a four‐group and five‐group trajectory model had fallen below 1%, we chose the four‐group trajectory model.

We named the groups (1) patient–caregiver low distress, (2) patient–caregiver increasing distress, (3) patient low–caregiver borderline distress and (4) patient–caregiver high distress. See Figure 2. Group 1 (patient–caregiver low distress; 27%) consisted of dyads with persistently low patient–caregiver distress (patient and caregiver mean HADS‐A and HADS‐D range between 1.05 and 3.70). Group 2 (patient–caregiver increasing distress; 28.5%) consisted of dyads where patients had increasing anxiety and depression (HADS‐A range = 3.17–6.07; HADS‐D range = 3.38–7.95), and their caregivers also reported increasing anxiety and depression (HADS‐A range = 4.38–8.20; HADS‐D range = 2.63–5.00). Group 3 (patient low–caregiver borderline distress; 25%) consisted of dyads where patients reported stable low distress (HADS‐A and HADS‐D range = 0.00–2.30) while caregivers reported persistent borderline distress where anxiety scores were elevated (HADS‐A range = 7.77–9.43) compared to depression scores (HADS‐D range = 5.46–7.83). Group 4 (patient–caregiver high distress; 19.5%) consisted of dyads where patients reported distress (HADS‐A range = 4.13–7.75; HADS‐D range = 5.31–10.56) in tandem with persistently high caregiver distress (HADS‐A range = 10.04–11.86; HADS‐D range = 7.89–10.40). The increases in psychological distress (HADS‐A and HADS‐D) scores for the patients in Groups 2 and 4 can be considered clinically meaningful base on past studies which found a minimal important difference range of between 1 and 1.8.^37^, ^38^, ^39^

### 3.3 Predictors of group membership

Table 2 shows the factors predicting group membership, using Group 1 (patient‐caregiver low distress) as the reference group. Dyads where the patient is below 50 years of age were more likely to be in Group 4 (patient‐caregiver high distress). We conducted some sensitivity analyses by examining the distribution of dyads with young patients among the four groups. We found that 47% of dyads with patients under the age of 50 and 56% of dyads with patients under the age of 40 were in Group 4.

**TABLE 2:** Predictors of trajectory group membership

	Joint trajectories						
	Group 1 PT‐CG low distress	Group 2 PT‐CG increasing distress		Group 3 PT low distress‐CG borderline distress		Group 4 PT‐CG high distress	
	[reference group]	Coef. (SE)	[95% CI]	Coef. (SE)	[95% CI]	Coef. (SE)	[95% CI]
Patient characteristics							
Age ≤ 49		0.97 (0.84)	[−0.68; 2.63]	1.22 (0.82)	[−0.39; 2.83]	2.73 (0.82)***	[1.11; 4.35]
Gender (male)		−0.06 (0.54)	[−1.12; 1.00]	0.55 (0.52)	[−0.48; 1.58]	0.04 (0.58)	[−1.10; 1.19]
Caregiver characteristics							
Age		0.00 (0.02)	[−0.04; 0.05]	−0.02 (0.02)	[−0.07; 0.02]	0.01 (0.03)	[−0.04; 0.06]
Gender (male)		0.48 (0.56)	[−0.62; 1.58]	0.11 (0.55)	[−0.96; 1.18]	0.60 (0.60)	[−0.56; 1.77]
Marital status (married)		−0.72 (0.65)	[−1.99; 0.55]	−0.32 (0.66)	[−1.61; 0.97]	−1.17 (0.72)	[−2.58; 0.24]
Employment status		0.19 (0.44)	[−0.67; 1.05]	1.07 (0.46)*	[0.17; 1.97]	0.48 (0.49)	[−0.49; 1.44]
Financial adequacy		−1.29 (0.49)**	[−2.24; −0.034]	−1.45 (0.47)**	[−2.38; −0.53]	−1.77 (0.50)***	[−2.75; −0.79]
Relationship to patient (ref: Spouse)							
Child		0.94 (0.68)	[−0.39; 2.28]	0.51 (0.67)	[−0.81; 1.83]	0.78 (0.79)	[−0.76; 2.32]
Others		0.43 (0.65)	[−0.85; 1.71]	−0.39 (0.72)	[−1.81; 1.03]	−0.71 (0.82)	[−2.33; 0.90]
Emotionally close to patient		−0.99 (0.37)**	[−1.71; −0.27]	−0.55 (0.38)	[−1.28; 0.19]	−0.92 (0.39)*	[−1.69; −0.15]

* *p* < 0.05

** *p* < 0.01

*** *p* < 0.001.

Dyads where caregiver‐patient emotional closeness was low were more likely to be in Group 2 or 4, where the patient and caregiver reported increasing/high distress. Dyads with caregivers who were employed were more likely to be in the Group 3 (patient low–caregiver borderline distress), while dyads where caregivers reported financial inadequacy were more likely to be in Groups 2–4.

## 4 DISCUSSION

The primary aim of the study was to examine the trajectories of psychological distress (anxiety and depressive symptoms) among advanced cancer patient–caregiver dyads across nearly three years. Joint trajectory group modelling revealed four distinct trajectory groups characterised by distinct anxiety and depressive patterns. Group 1 (27% of sample) were the best‐faring group with consistently low‐dyad distress over time, while Group 4 (19.5%) were the worst off, with high dyad distress over time. Almost half of the dyads in the study had both patient and caregiver reported increasing or high anxiety and/or depression scores over time (Groups 2 and 4).

Among patients, we found that approximately 1 out of 4 reported increasing levels of depression (Group 2), and 1 out of 5 increasing levels of anxiety (Group 4) over 33 months. This may be because patients' advancing disease which is often associated with worsening physical symptoms—consequently leading to increased anxiety and depression. Nearly half the patients in our sample report either increasing depression/anxiety levels over time (Groups 2 and 4), where the score change is considered clinically meaningful.^37^, ^38^, ^39^ Our findings underscore the importance of continued monitoring of mood symptoms over time as detection can lead to timely intervention to improve patient outcomes.

Results further demonstrated that a substantial proportion (approximately 45%) of caregivers that comprised of Group 3 and Group 4 reported distress that were consistently higher compared to the patients they were caring for. Our findings are consistent with a prior German cross‐sectional study where trends of higher distress scores among caregivers compared to patients were reported.^8^ This may be because while both dyadic members experience cancer‐related fears, caregivers typically also have other responsibilities including the care of patient, other dependents (e.g. children and grandchildren), career‐related responsibilities and providing financial support.^40^, ^41^ Caring for loved ones who are ill is also more often expected in the Asian context. While filial piety is an important tenet of our culture, it can also increase feelings of social pressure.^42^

Results also indicated that dyads that included patients younger than 50 years old were more likely to be in the group with the highest distress. Our sensitivity analyses subsequently corroborated that about approximate half of sample participants under 40 or 50 years of age fell into this group. This is consistent with literature demonstrating that younger adults diagnosed with advanced stage cancer are at higher risk for distress,^20^ and underscores how particularly vulnerable they and their caregivers may be.

Though literature regarding the associations between emotional closeness and dyadic psychological well‐being remain mixed, our studies are consistent with a prior study among caregivers of dementia patients, where higher emotional closeness was associated with better mental health (i.e. lower depression scores).^31^ We found that dyads where caregiver–patient emotional closeness was low were more likely to be in Group 2 or 4 where patients and caregivers reported increasing/high distress. It is possible that without a close emotional bond, providing care for individuals over extended periods was unfulfilling and frustrating, exacerbating feelings of burden and distress. This would be consistent with some of our prior work that suggests sense of meaning is associated with caregiving outcomes.^43^

Caregiver‐reported inadequate finances were also found to be associated with subgroups where dyads reported distress (Groups 2– 4). While MediSave (a national medical savings scheme) can be used to subsidise medical expenses in Singapore, other costs of healthcare—particularly for severe, chronic and expensive conditions such as cancer—can remain cost‐prohibitive. Though recent government resources such as the Cancer Care Fund^44^, ^45^ and the Medication Assistance Fund have been enacted to alleviate the financial burden of low‐to‐middle income families, these may not be enough to relieve the financial burden felt by caregivers. This is an important issue given recent evidence indicating that financial toxicity can worsen multiple health dimensions including subjective well‐being, health‐related quality of life, mortality and quality of care.^46^

We also found that dyads with caregivers who were employed (including full‐ or part‐time) were more likely to be in Group 3, where patients reported low distress and caregiver reported borderline levels of distress. It is possible that juggling both work and caregiving duties, or working and feeling less able to meet patient needs, can result in increased caregiver distress. For this group of dyads, caregivers can perhaps be connected to resources (e.g. nursing care, palliative home‐based care) and social and governmental cancer support networks (e.g. Ains Society^47^ and Buddies of NCCS^48^ in Singapore) to help meet their needs.

### 4.1 Clinical implications

Our findings suggest that to improve the psychological well‐being of patient‐caregiver dyads, greater efforts should be directed towards the intermittent screening of both members for distress. While initial efforts to monitor cancer patients have begun, caregivers are still not monitored in any systematic manner despite the prevalence of caregiver‐reported distress.^49^ Beyond depending on self‐report tools, it may be important for oncologic healthcare teams to keep in mind psychosocial risk factors for dyadic distress: young patient age, financial difficulties, caregivers who are working and when the relationship between patient‐caregiver are not close or strong.

Subsequently, resources for practical aid (e.g. financial assistance, counselling, home nursing care) should be allocated for caregivers. The allocation of resources by healthcare institutions to reduce caregiver burden is not only helpful for dyadic psychological health, but also makes pragmatic sense—improving the psychological well‐being of the caregiver will lead to increased quality of care for patients.^50^

### 4.2 Limitations and future research

There are several limitations to be mentioned. Our study focus was on advanced cancer patient–caregiver dyads recruited through convenience sampling from two public Singapore hospitals. Our results may not be generalisable to other serious illnesses, or different socioeconomic and cultural settings. Additionally, because data were analysed according to time from study enrolment, patients and caregivers may have been at different stages of their terminal illness that affect their psychological distress.

### 4.3 Study strengths/conclusions

There are two main strengths to this study. First, its usage of group trajectory modelling to examine the joint trajectories of psychological distress among advanced cancer dyads for nearly three years allowed a better understanding of the intricate relationships between the psychological well‐being of caregivers and patients, and subsequently identifying risk factors for dyadic distress that provides  a framework for developing interventions focusing on both dyadic members. Second, we also focused on an Asian country where there is still limited data, and where we expect cultural expectations surrounding caregiving (i.e. filial piety) to influence dyadic well‐being. In conclusion, there is heterogeneity in how dyadic members experience psychological distress over time, and healthcare providers must consider dyadic differences when formulating interventions. Resources should be allocated to identifying patient and caregivers who can benefit from psychological support during patient's end‐of‐life care.

## AUTHOR CONTRIBUTIONS

**Irene Teo:** Conceptualization (lead); formal analysis (equal); methodology (equal); supervision (equal); writing – original draft (equal); writing – review and editing (equal). **Sean Ng:** Formal analysis (equal); writing – original draft (equal); writing – review and editing (equal). **Filipinas Gines Bundoc:** Formal analysis (equal); writing – review and editing (equal). **Chetna Malhotra:** Writing – review and editing (equal). **Semra Ozdemir:** Writing – review and editing (equal). **Jennifer L Steel:** Writing – review and editing (equal). **Eric Andrew Finkelstein:** Supervision (equal); writing – review and editing (equal). **COMPASS Group:** Data curation (equal); project administration (equal).

## FUNDING INFORMATION

The study is funded by Singapore Millennium Foundation (2015‐SMF‐0003) and Lien Centre for Palliative Care (LCPC‐IN14‐0003).

## CONFLICT OF INTEREST STATEMENT

The authors declare no conflicts of interest.

## ETHICS APPROVAL STATEMENTS

Both patients and their caregivers who agreed to participate provided written informed consent. The COMPASS study was approved by SingHealth Centralised Institutional Review Board (2015/2781).

## Supporting information


**Supporting information S1.** Supplementary material

## Data Availability

The data that support the findings of this study are available from the corresponding author upon reasonable request.
