# Supplementary material for: A prospective study of psychological distress among patients with advanced cancer and their caregivers
Source: Cancer Med. 2023 Mar 19;12(8):9956–65. doi: 10.1002/cam4.5713 (PMC10166955; doi:10.1002/cam4.5713)
Supplement: Supplementary file 1 — Supporting information S1. Supplementary material [file CAM4-12-9956-s001.docx]

**Supplementary Figure 1. COMPASS participant flow diagram**

Approached patients (n=1,137)

Excluded (n=95)

- Did not meet inclusion criteria (n=95)

Patient-Caregiver Dyad Analysed (n=299)

Excluded (n=301)

- Patients without caregiver (n=289)
- Patient and caregivers who answered each survey more than 14days apart (n=12)

Patients with baseline data (n=600)

Excluded (n=49)

- Withdrew consent before completion of baseline (n=2)
- Records review only (n= 47)

Declined to participate (n=393)

- Not interested/ Indecisive (n=306)
- No time to participate (n=4)
- Research fatigue (n=20)
- Too ill or has hearing, vision, or speech impairment (n=34)
- Caregiver rejected (n=29)

Eligible patients (n=1,042)

Enrolled patients(n=649)

***Supplementary Table* 1. Frequency counts and percentages of patients who passed on from baseline up to month 33**

| **Patients who died in or before:** | **Frequency (%)** | **Sample size** |
| --- | --- | --- |
| Month 0 | **-** | 299 |
| Month 3 | 12 (4%) | 287 |
| Month 6 | 27 (9%) | 260 |
| Month 9 | 28 (9%) | 232 |
| Month 12 | 21 (7%) | 211 |
| Month 15 | 17 (6%) | 194 |
| Month 18 | 22 (7%) | 172 |
| Month 21 | 16 (5%) | 156 |
| Month 24 | 9 (3%) | 147 |
| Month 27 | 10 (3%) | 137 |
| Month 30 | 8 (3%) | 129 |
| Month 33 | 6 (2%) | 123 |
|  |  |  |
| **Total** | **176 (58%)** |  |

***Supplementary Table* 2. Summary statistics of trajectory groups based on the number of trajectory group memberships**

| **Number of trajectory groups** | **Trajectory group** | **Group BIC** | **% Change in BIC** | **Percentage of group membership** | **Group average posterior probability (APP)** |
| --- | --- | --- | --- | --- | --- |
| 1 | 1 | -17689.2 | - | 100.00 | 1 |
| 2 | 1 | -16946.64 |  | 54.76 | 0.96 |
|  | 2 |  | 4.38% | 45.24 | 0.95 |
| 3 | 1 | -16635.14 |  | 40.82 | 0.94 |
|  | 2 |  | 1.87% | 29.60 | 0.91 |
|  | 3 |  |  | 29.59 | 0.93 |
| 4 | 1 | -16435.57 |  | 26.97 | 0.92 |
|  | 2 |  |  | 28.51 | 0.86 |
|  | 3 |  | 1.21% | 25.06 | 0.92 |
|  | 4 |  |  | 19.46 | 0.95 |
| 5 | 1 | -16290.24 |  | 12.30 | 0.88 |
|  | 2 |  |  | 20.11 | 0.85 |
|  | 3 |  | 0.89% | 30.33 | 0.90 |
|  | 4 |  |  | 17.60 | 0.94 |
|  | 5 |  |  | 19.66 | 0.90 |
|  |  |  |  |  |  |
